# Neuromuscular Evaluation in Orthodontic–Surgical Treatment: A Comparison Between Monomaxillary and Bimaxillary Surgery

**DOI:** 10.3390/bioengineering13010123

**Published:** 2026-01-21

**Authors:** Lucia Giannini, Luisa Gigante, Giada Di Iasio, Giovanni Cattaneo, Cinzia Maspero

**Affiliations:** 1 Fondazione IRCCS Cà Granda Ospedale Maggiore Policlinico, 20122 Milan, Italy; giada.diiasio@unimi.it (G.D.I.);; 2 Department of Biomedical, Surgical and Dental Sciences, School of Dentistry, University of Milan, 20122 Milan, Italygiovanni.cattaneo1@unimi.it (G.C.)

**Keywords:** orthognathic surgery, surface electromyography, monomaxillary surgery, bimaxillary surgery, mandibular kinesiography

## Abstract

Purpose: Orthognathic surgery is a cornerstone therapeutic approach for correcting dentofacial deformities; however, its Impact on neuromuscular adaptation remains incompletely understood, particularly regarding different surgical strategies. The aim of this study was to evaluate and compare neuromuscular changes in patients undergoing monomaxillary or bimaxillary orthognathic surgery. Methods: Eighty adult patients treated with combined orthodontic–surgical therapy were included (37 monomaxillary; 43 bimaxillary). A control group of 20 healthy adult subjects with physiological occlusion and no history of orthodontic or orthognathic treatment was included. Surface electromyography (sEMG) of the masseter and anterior temporalis muscles and mandibular kinesiography were performed using standardized protocols at five treatment phases. Electromyographic symmetry indices (Percent Overlapping Coefficient—POC), muscle activity (µV), IMPACT values, and mandibular movement parameters were analyzed. Results: During the presurgical orthodontic phase, both groups showed comparable reductions in neuromuscular activity. Postoperatively, monomaxillary patients exhibited earlier stabilization of sEMG symmetry and a faster increase in IMPACT values, approaching physiological reference ranges at the final follow-up. In contrast, bimaxillary patients showed greater variability and slower functional recovery. Mandibular opening and lateral movements improved in all patients, with more stable kinesiographic patterns observed in the monomaxillary group. Conclusions: Within the limitations of this study, neuromuscular adaptation following orthodontic–surgical treatment appears to be associated with the surgical approach adopted, rather than representing a direct effect of surgical extent. These findings support the role of functional assessment as a complementary component in the management of orthognathic patients.

## 1. Introduction

Orthognathic surgery represents a cornerstone approach in the management of dentofacial deformities, enabling the correction of skeletal discrepancies that cannot be resolved through orthodontics alone. By repositioning the skeletal bases, this treatment improves occlusion, masticatory function, and facial aesthetics, while providing indirect benefits to breathing and phonation, ultimately improving patient quality of life [1,2,3,4]. Within this framework, craniofacial morphology is understood as the result of a complex interaction between genetic, environmental, and functional factors, where the neuromuscular system plays a pivotal role [5,6,7,8,9].

Alterations in muscle function and occlusal forces can significantly influence dentoalveolar development and the stability of skeletal relationships across all three planes of space [6,7,8,9]. Although numerous studies have investigated the relationship between temporomandibular joint (TMJ) function, masticatory muscles, and craniofacial morphology, limited literature has systematically evaluated neuromuscular changes induced specifically by orthodontic–surgical treatment [10,11,12,13,14,15,16,17,18].

Surface electromyography (sEMG) is a non-invasive method for assessing muscle function by analyzing the intensity, coordination, and asymmetry of masticatory muscles [19,20,21]. However, findings regarding the association between sEMG patterns and skeletal phenotypes remain inconsistent. Regarding the vertical dimension, some authors report reduced muscular efficiency in subjects with a high mandibular plane angle, whereas patients with a deep bite tend to exhibit higher masticatory muscle activity compared to those with an open bite [22,23,24,25,26]. Farronato et al. demonstrated significant differences in EMG patterns between open bite and deep bite patients, emphasizing the crucial role of the neuromuscular component in treatment stability [27]. Similar considerations apply to the sagittal plane, where distinct neuromuscular patterns in Class II and Class III subjects reflect differences in muscular recruitment and functional adaptation.

In addition to sEMG, kinesiographic analysis of mandibular movements is an essential tool for an integrated functional evaluation. The integration of EMG and mandibular kinesiography allows for the correlation of masseter and anterior temporalis activity with kinematics—including mouth opening, protrusion, and lateral movements [27]. This combined approach also allows researchers to assess the influence of skeletal repositioning (monomaxillary vs. bimaxillary) on functional recovery and the persistence of compensatory patterns.

While orthodontic–surgical treatment aims to achieve a physiological configuration, it inevitably induces functional reorganization [28,29,30,31]. During the presurgical phase, a reduction in maximum bite force and mandibular excursion is often documented, likely reflecting neuromuscular protective mechanisms and transient occlusal interferences [32,33,34]. Postoperatively, inflammation and edema may further reduce function and prolong recovery [35]. Longitudinal studies show that sEMG activity tends to decrease in early postoperative phases before gradually recovering; however, this recovery does not always reach the values observed in subjects with physiological occlusion [36].

A major limitation in the current literature is the lack of focus on the specific type of surgical intervention. Most studies analyze heterogeneous samples, neglecting the potential differential Impact of monomaxillary versus bimaxillary surgery [37]. It is hypothesized that different degrees of skeletal repositioning result in distinct functional responses. Therefore, this study aims to compare neuromuscular changes in patients undergoing monomaxillary and bimaxillary surgery using a standardized electromyographic and electrokinesiographic approach.

## 2. Materials and Methods

### 2.1. Study Sample

The present study included a cohort of 80 adult patients (≥18 years) who underwent combined orthodontic–surgical treatment at the Department of Orthodontics, University of Milan. The sample consisted of patients presenting with dentoskeletal discrepancies requiring orthognathic surgery. According to the surgical approach, 43 patients underwent bimaxillary surgery, while 37 patients were treated with monomaxillary surgery. Within the monomaxillary cohort, 22 patients underwent maxillary surgery, while the others received mandibular surgery alone. The allocation of patients to the monomaxillary or bimaxillary surgery group was based exclusively on clinical, skeletal, and aesthetic indications. Therefore, the surgical approach was not treated as an independent variable, and no matching was performed for skeletal classification, severity of deformity, or magnitude and direction of skeletal displacement.

### 2.2. Inclusion Criteria

1. End of craniofacial growth.
2. Presence of a dentoskeletal discrepancy requiring combined orthodontic–surgical treatment.
3. Full compliance with the electromyographic and kinesiographic protocol.

### 2.3. Exclusion Criteria

1. Presence of systemic or neuromuscular disorders at the beginning of treatment.
2. Ongoing craniofacial growth.
3. Incomplete participation in the scheduled functional examinations.

A control group of 20 healthy adult subjects with physiological occlusion and no history of orthodontic or orthognathic treatment was included. The control group was used to provide reference values for electromyographic and kinesiographic parameters and to allow comparison with neuromuscular patterns observed in patients at baseline and during follow-up. The control group was included to provide reference values for physiological neuromuscular patterns. Control data were not used for formal equivalence testing but served as a descriptive benchmark to contextualize patient values at baseline and follow-up.

All patients provided informed consent prior to participation. The functional examinations performed were non-invasive, and the study was approved by the Ethics Committee (protocol code 421, date of approval 9 March 2016).

### 2.4. Sample-Size

An a priori sample-size calculation was performed to determine the minimum number of subjects required to detect clinically meaningful differences in electromyographic parameters between the monomaxillary and bimaxillary groups. Based on data from previous studies on neuromuscular outcomes after orthognathic surgery and assuming a medium effect size, a significance level of 0.05 and a statistical power of 80%, the estimated required sample size was approximately 70 patients. To account for potential missing data and incomplete longitudinal records, a total sample of 80 patients was included in the present study. This sample size was therefore considered adequate to ensure sufficient power for the primary comparative analyses between the two surgical approaches. This power estimation refers only to the primary descriptive contrasts between the monomaxillary and bimaxillary groups and is not intended to power longitudinal inferential comparisons.

### 2.5. Recordings

All patients underwent periodic surface electromyographic (sEMG) evaluations of the masticatory muscles (masseter and temporal muscles) and electrokinesiographic analysis of mandibular movements, following standardized protocols [26,27,28].

Surface electromyography was performed on the anterior temporal and superficial masseter muscles, bilaterally, using two different electromyographic systems:

- Freely electromyograph (De Gotzen, Legnano, Italy);
- K6-I electromyograph (Myotronics, Tukwila, WA, USA).

**Figure 1:**
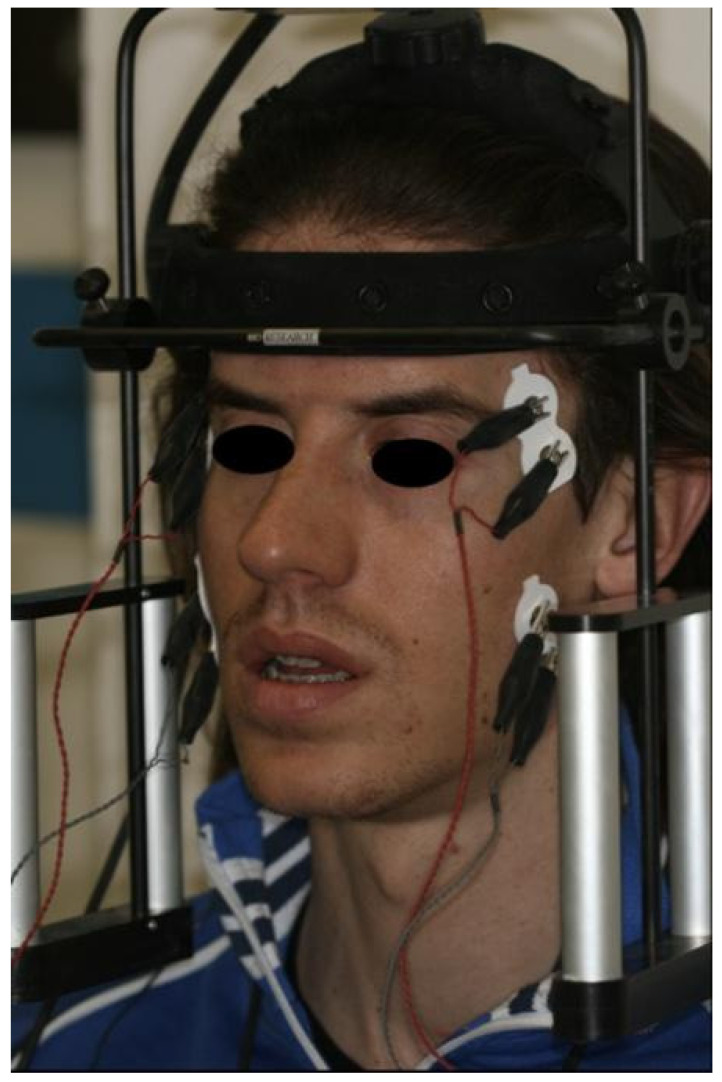
Positioning of bipolar surface electrodes for surface electromyography (sEMG) recording of the masticatory muscles. Electrodes were placed bilaterally over the anterior temporalis and superficial masseter muscles according to standardized anatomical landmarks, with alignment along muscle fiber orientation. A reference electrode was positioned on a neutral area of the forehead.

**Figure 2:**
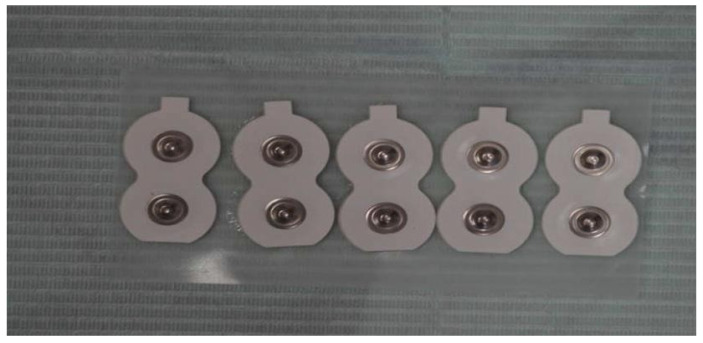
Disposable bipolar surface electrodes used for sEMG recordings. The inter-electrode distance and adhesive backing allow stable signal acquisition and reduction in motion artifacts during functional tasks.

Disposable bipolar surface electrodes were positioned after careful skin cleansing with a chlorhexidine–alcohol solution to reduce impedance (Figure 1). Electrode placement followed anatomical landmarks to ensure alignment with muscle fiber orientation, and a grounding electrode was positioned on a neutral area of the forehead [26,27,28] (Figure 2).

The placement of the electrodes followed the specified protocol:

- Masseter muscle: Staying behind the seated patient, the operator touched the muscle mass while the patient clenched the teeth. To position the bipolar electrode parallel to the muscle fibers, a line connecting the labial commissure to the tragus and a second line following the exocanthion–gonion axis were drawn. The electrode was positioned so that its superior pole lay at the intersection of the two lines, with its major axis aligned along the exocanthion–gonion line.
- Temporal muscle: The muscle mass was palpated while the patient clenched the teeth to identify the major axis of the zygomatic process of the frontal bone. The bipolar electrode was then positioned along a line parallel to this axis. In this manner, the electrode was aligned parallel to the muscle fibers and placed at a relatively superficial level with respect to the frontoparietal suture.

All patients underwent both surface electromyographic (sEMG) evaluations of the masticatory muscles and electrokinesiographic (EKG) analysis of mandibular movements at each therapeutic time point, following standardized protocols.

**Figure 3:**
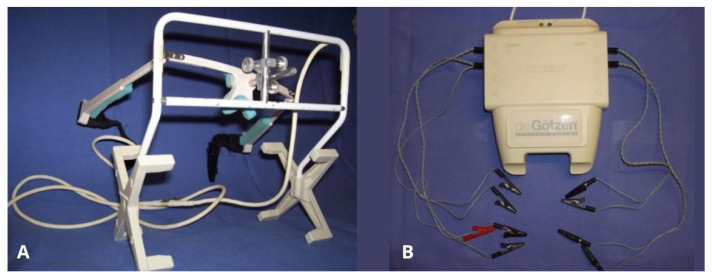
Electromyographic and electrokinesiographic recording systems used in the study. (**A**) K6-I system (Myotronics, Tukwila, WA, USA), used for recording raw electromyographic activity expressed in microvolts and for electrokinesiographic analysis of mandibular movements. (**B**) Freely electromyograph (De Gotzen, Legnano, Italy), used for standardized electromyographic indices such as Percent Overlapping Coefficient (POC) and IMPACT values.

**Figure 4:**
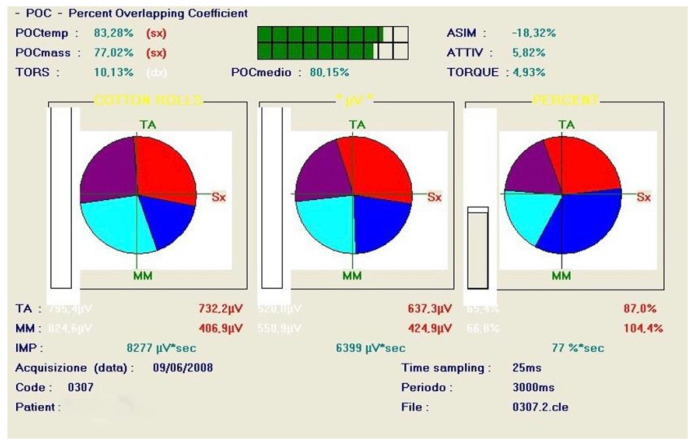
Example of Percent Overlapping Coefficient (POC) analysis obtained with the Freely system. POC quantifies the symmetry and coordination of bilateral masseter and anterior temporalis muscle activity during clenching tasks, expressed as a percentage of signal overlap between right and left muscles.

The two different electromyographic systems were used in a complementary and standardized manner, according to their technical specifications (Figure 3). The Freely electromyograph (De Gotzen, Legnano, Italy) was used to assess standardized electromyographic indices, including Percent Overlapping Coefficient (POC) and IMPACT values, which are based on normalized signal processing and are not dependent on raw signal amplitude (Figure 4). The K6-I system (Myotronics, Tukwila, WA, USA) was used to record electromyographic activity expressed in microvolts (µV) and to perform electrokinesiographic (EKG) analysis of mandibular movements, which cannot be evaluated using the Freely system.

### 2.6. Timing of Evaluations

Functional recordings were performed in five treatment phases:

- Initial diagnostic evaluation (T0, at baseline before any orthodontic treatment);
- Presurgical orthodontic phase (T1, at the end of presurgical orthodontics);
- Immediate postoperative phase (T2, 3/5 days post surgery);
- Postsurgical orthodontic phase (T3, during postsurgical orthodontic finishing—2 months post surgery);
- At the end of the orthodontic treatment (T4, at the end of treatment)
- Follow-up after completion of orthodontic–surgical treatment (2 years after the end of the treatment)

All examinations were carried out in a quiet room, with patients seated in a standardized upright position and natural head posture.

The analyzed parameters included electromyographic symmetry indices (POC, percent overlapping coefficient) of the masseter and anterior temporalis muscles, muscle activity expressed in microvolts, and IMPACT values recorded during clenching on cotton rolls and in habitual position. Electrokinesiographic parameters included maximum mouth opening, protrusion, lateral excursions, and mandibular rest position.

Each patient was consistently assessed using the same device for the same type of measurement at all treatment phases, ensuring intra-subject consistency across the longitudinal evaluations. As the two systems were used for different outcome domains and not for interchangeable measurements of the same variables, cross-calibration between devices was not required.

### 2.7. Recording Protocol

Electromyographic recordings were obtained during:

▪ Maximum voluntary clench (MVC) on cotton rolls placed on the posterior teeth (cotton clench);
▪ Maximum voluntary clench in habitual intercuspation (clench).

Each recording lasted 5 s and was repeated at least twice to ensure reliability, with only reproducible signals included in the analysis. Resting muscle activity was also recorded to assess baseline neuromuscular conditions.

**Figure 5:**
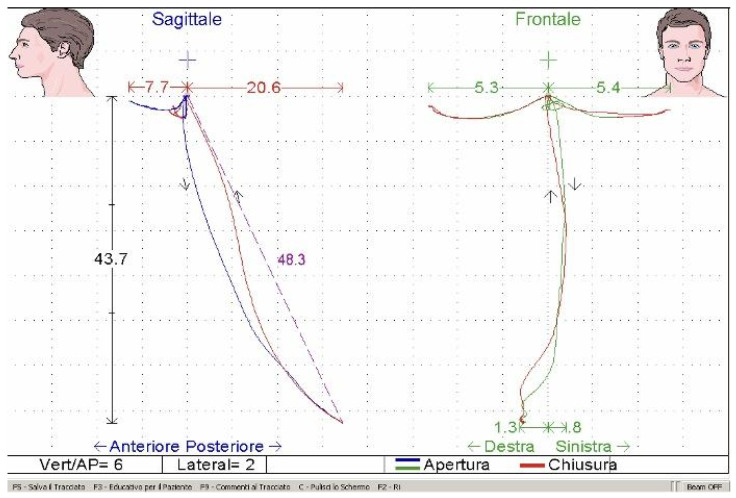
Electrokinesiographic recording of mandibular movements. Sagittal and frontal tracings illustrate maximum mouth opening, protrusion, and lateral excursions during functional tasks, allowing quantitative assessment of mandibular kinematics throughout treatment phases.

Electrokinesiographic evaluation included the analysis of mandibular movements such as maximum opening, protrusion, lateral excursions, mandibular rest position, and freeway space (Figure 5). When indicated, transcutaneous electrical nerve stimulation (TENS) was applied to evaluate post-relaxation neuromuscular behavior, following standardized procedures. To minimize variability, analyses focused on standardized indices rather than raw signal amplitude.

### 2.8. Statistical Analysis

Data were analyzed using Student’s *t*-test for independent samples to compare monomaxillary and bimaxillary groups at each evaluation time point. The level of statistical significance was set at *p* < 0.05. Continuous variables were expressed as mean ± standard deviation. Normality of data distribution was assessed using the Shapiro–Wilk test. Comparisons between each surgical group and the control group were performed using independent-sample *t*-tests to evaluate differences from physiological reference values at baseline and at final follow-up. Statistical analyses were performed using SPSS software (version 28.0.0; IBM Corp., Armonk, NY, USA).

Given the exploratory nature of the study and its focus on longitudinal neuromuscular behavior across predefined treatment phases, statistical results were interpreted in light of their clinical relevance and temporal consistency; therefore, no adjustment for multiple comparisons was applied.

Given the observational design of the study and the absence of matching for skeletal characteristics, statistical comparisons were intended to describe associative differences between groups rather than to infer causal relationships. The applied statistical approach was intended to provide descriptive comparisons between groups at each clinically relevant time point. This strategy does not model intra-subject correlation or formal time × group interactions; therefore, results should be interpreted as associative and descriptive longitudinal patterns rather than inferential temporal effects.

Subgroup analyses comparing maxillary-only and mandibular-only procedures were performed on an exploratory basis and were not included in the original sample size calculation. These analyses were intended to generate hypotheses rather than to provide confirmatory evidence.

Comparisons with the control group were intended to provide descriptive reference ranges rather than to establish functional equivalence.

## 3. Results

The analysis of the data obtained from this study evidenced differences in neuromuscular behavior according to the surgical approach adopted.

**Figure 6:**
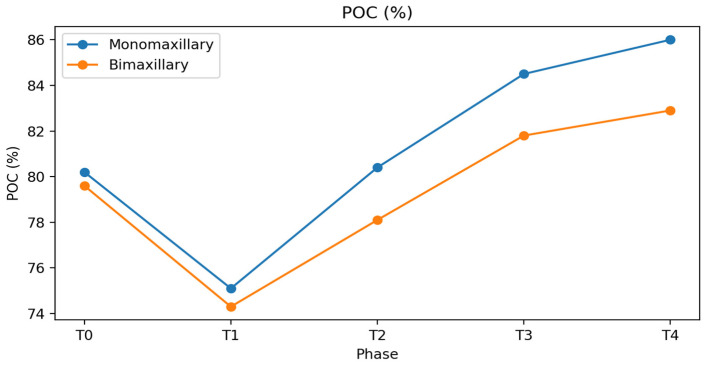
Percent Overlapping Coefficient (POC, %) of masseter and anterior temporalis muscles in monomaxillary and bimaxillary patients across treatment phases (T0–T4). Values are expressed as mean ± standard deviation. The graph illustrates longitudinal trends only. Asterisks in the table indicate between-group differences at the same treatment phase (*p* < 0.05).

With regard to electromyographic symmetry, Percent Overlapping Coefficient (POC) values of the masseter and anterior temporalis muscles, as well as mean POC, showed slight differences between monomaxillary and bimaxillary patients throughout the treatment phases (Table 1). At baseline (T0), mean POC values were comparable between groups (monomaxillary: 80.2 ± 3.5%; bimaxillary: 79.6 ± 3.8%). During the presurgical orthodontic phase, both groups showed a reduction in symmetry (75.1 ± 4.0% and 74.3 ± 4.2%, respectively). Only a limited number of differences reached statistical significance, mainly during the postsurgical and follow-up phases. From the immediate postsurgical phase, monomaxillary patients exhibited higher and more stable POC values (80.4 ± 3.6%) compared with bimaxillary patients (78.1 ± 4.1%), with a further increase at final follow-up (86.0 ± 3.2% vs. 82.9 ± 3.9%, *p* < 0.05), indicating a pattern of neuromuscular activity associated with greater symmetry, without implying a direct effect of the surgical approach (Table 1 and Figure 6). POC values observed at follow-up were comparable to those of the control group (85 ± 1.2). The group treated with the monomaxillary approach reached and exceeded this reference value, whereas the bimaxillary group approached it closely without fully attaining it, numerically lower than references values.

The IMPACT index is an electromyographic parameter that quantifies the overall muscular workload generated by the masticatory muscles during maximum voluntary clenching. It is calculated as the integrated area under the EMG signal curve over time, and it reflects both the intensity and the duration of muscle activation.

At baseline (T0), both monomaxillary and bimaxillary patients generally exhibit lower values compared with healthy controls (6100 ± 800 µV·s), reflecting reduced functional efficiency but the presence of a compensatory equilibrium to the skeletal malocclusion.

At baseline, Impact values were approximately 5000 ± 800 µV·s in both groups and decreased to about 4000 ± 700 µV·s in the preoperative phase. In the immediate postsurgical phase, a marked reduction was observed, more pronounced in bimaxillary patients (2000 ± 500 µV·s) than in monomaxillary patients (2500 ± 600 µV·s). During the postsurgical orthodontic phase, Impact values increased more rapidly in monomaxillary patients—particularly in those treated with maxillary surgery alone—reaching values closer to physiological ranges at the end of treatment.

**Figure 7:**
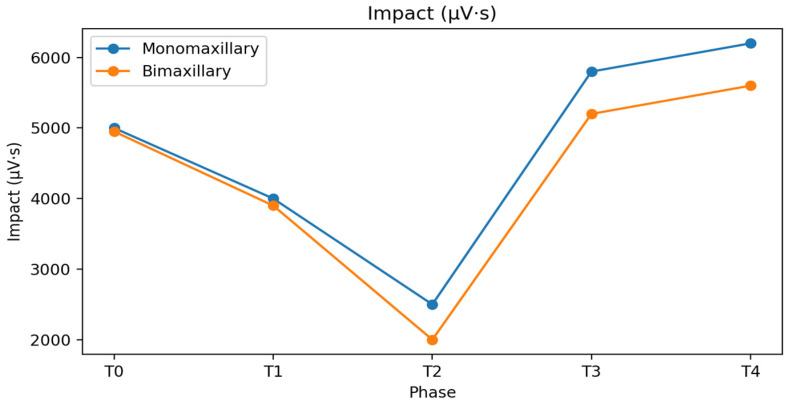
Impact values (µV·s) of masticatory muscles in monomaxillary and bimaxillary patients throughout the orthodontic–surgical treatment. Data are reported as mean ± standard deviation.

At final follow-up, Impact values in the monomaxillary group (6200 ± 900 µV·s) were significantly higher than those observed in the bimaxillary group (5600 ± 850 µV·s, *p* < 0.05), suggesting a more efficient functional recovery. (Table 2 and Figure 7). The IMPACT values observed at follow-up were comparable to those of the control group (6100 ± 800). The group treated with the monomaxillary approach reached and exceeded this reference value, whereas the bimaxillary group closely approached it without fully achieving it, remaining numerically lower than the reference values. 

**Figure 8:**
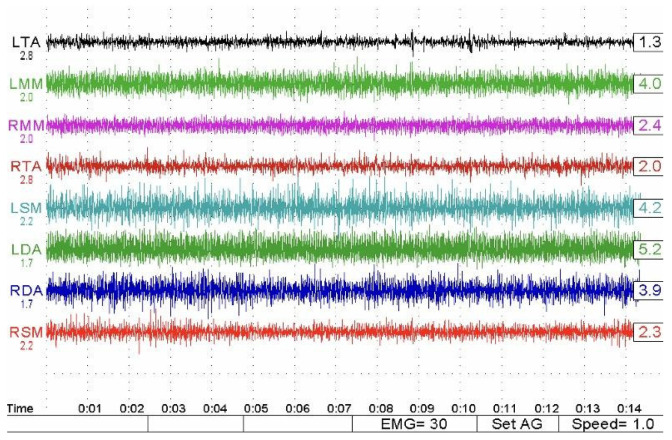
Representative surface electromyographic (sEMG) recordings showing masticatory muscle activity expressed in microvolts (µV). Tracings illustrate bilateral activity of the anterior temporalis and masseter muscles during clenching tasks, highlighting inter-muscular coordination and amplitude changes across treatment phases.

Muscular activity expressed in microvolts was comparable between groups at baseline (approximately 5000 ± 750 µV in both groups) and showed a transient reduction after the initiation of orthodontic therapy (about 4000 ± 700 µV) (Figure 8).

**Figure 9:**
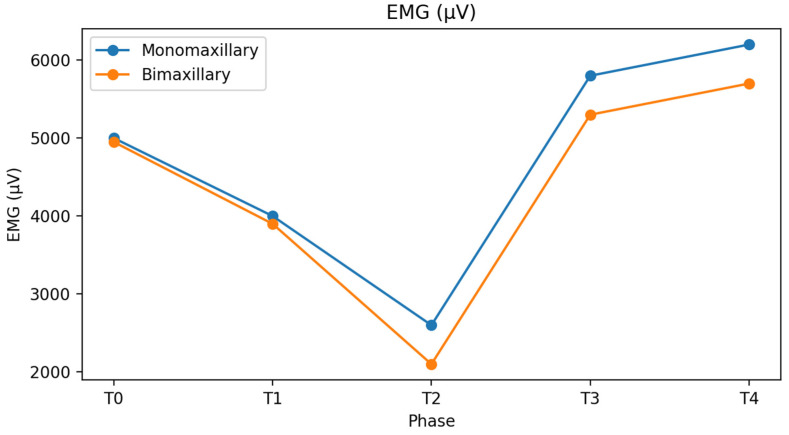
Mean electromyographic activity (µV) of masticatory muscles recorded during clenching on cotton rolls and habitual intercuspation in monomaxillary and bimaxillary patients at each treatment phase.

No statistically significant differences were observed in the magnitude of this initial reduction between monomaxillary and bimaxillary patients (Table 3). In the immediate postsurgical phase, values further decreased (monomaxillary: 2600 ± 600 µV; bimaxillary: 2100 ± 550 µV) and then progressively increased in all groups. However, monomaxillary patients, particularly those treated on the upper jaw, demonstrated a faster and more homogeneous recovery, reaching mean values of about 6200 ± 850 µV at final follow-up, whereas bimaxillary patients showed lower mean values (5700 ± 800 µV) and greater interindividual variability. This pattern was evident both during clenching on cotton rolls and clenching in habitual intercuspation, with no relevant differences related to the type of clench (Figure 9). The muscle activity values (expressed in µV) observed at follow-up approached those of the control group (6300 ± 700) in both treated groups. However, while both groups showed a clear tendency toward normalization, the bimaxillary group continued to show values that remained numerically lower than the reference values of the control group.

Subgroup analysis within the monomaxillary cohort revealed further differences. Patients treated with maxillary surgery alone showed higher POC (87.1 ± 3.0% vs. 84.2 ± 3.5%) and Impact values (6400 ± 850 µV·s vs. 5800 ± 800 µV·s) and a more rapid normalization of muscular activity compared with patients treated with mandibular surgery alone. In contrast, mandibular-only procedures were associated with a slower recovery pattern and greater variability in neuromuscular parameters during follow-up (Figure 9).

**Figure 10:**
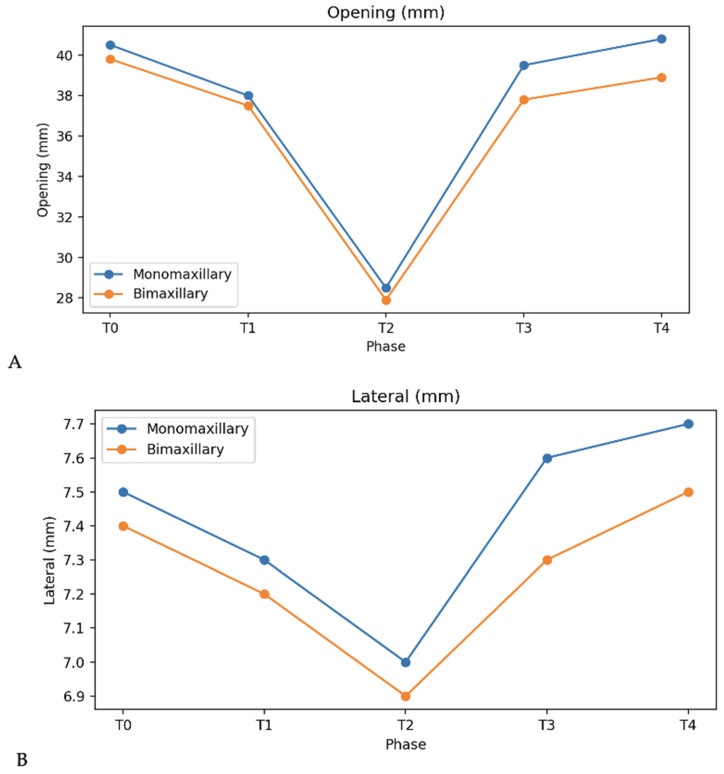
Mandibular kinesiographic parameters (mm) recorded in monomaxillary and bimaxillary patients during the observation period: (**A**) maximum mouth opening; (**B**) lateral excursions.

Regarding mandibular kinesiography, maximum mouth opening did not differ significantly between groups at baseline (monomaxillary: 40.5 ± 4.2 mm; bimaxillary: 39.8 ± 4.5 mm) (Figure 10). In the immediate postsurgical phase, mouth opening decreased in both groups, reaching values below 30 mm (28.5 ± 3.8 mm and 27.9 ± 4.0 mm, respectively). Subsequently, a progressive recovery was observed, with monomaxillary patients demonstrating a more stable improvement, returning to values close to baseline at the end of treatment (40.8 ± 4.0 mm), whereas bimaxillary patients showed slightly lower values (38.9 ± 4.3 mm). Lateral mandibular movements showed a similar pattern, with progressive improvement after surgery and no statistically significant differences between groups at final follow-up.

No statistically significant differences were observed between groups for mandibular lateral movements at any treatment phase, with mean values remaining stable around 7–8 mm throughout the observation period (Figure 10).

At the final follow-up, no differences were observed between patient groups and the control group; however, this finding should be interpreted as an approximation toward physiological reference values rather than formal normalization.

With regard to mouth opening and lateral movements, the observed differences among groups were minimal and, even when statistically significant, remained within the limits of normal functional variability.

## 4. Discussion

The relationship between craniofacial morphology, occlusal function, and neuromuscular behavior has long been recognized as complex and multifactorial, involving adaptive mechanisms that develop throughout growth and treatment [2,3,6]. Orthognathic surgery represents a major functional perturbation of this system, and its effects on masticatory muscle activity have been widely investigated, although with heterogeneous and sometimes conflicting results [4,5,28,29,30].

The present study investigated neuromuscular behavior during orthodontic–surgical treatment, focusing on the influence of the surgical approach (monomaxillary versus bimaxillary surgery) on electromyographic and kinesiographic parameters. The findings suggest that different patterns of neuromuscular adaptation may be associated with the extent of surgical intervention; however, these observations should be interpreted as associations rather than causal effects.

The comparison between monomaxillary and bimaxillary surgery groups should be interpreted within the context of an observational clinical design. Since the surgical approach is determined by anatomical, skeletal, and aesthetic requirements, it cannot be considered an independent variable. Consequently, the differences observed in neuromuscular behavior reflect associative patterns rather than direct effects attributable solely to the extent of surgical intervention.

Consistent with previous reports, both monomaxillary and bimaxillary patients exhibited a reduction in muscle activity, symmetry indices, and Impact values during the presurgical orthodontic phase [32,33,34]. This transient neuromuscular deterioration is commonly attributed to orthodontic decompensation, which disrupts pre-existing dentoalveolar compensatory mechanisms and temporarily compromises occlusal stability [6,10,11]. Importantly, the magnitude of this reduction was comparable between groups, suggesting that presurgical orthodontics imposes a similar functional burden regardless of the subsequent surgical strategy.

Following surgery, however, differences in neuromuscular behavior emerged between the two groups. Patients treated with monomaxillary surgery showed a tendency toward earlier stabilization of electromyographic symmetry, although only some differences reached statistical significance, as reflected by higher and more consistent Percent Overlapping Coefficient (POC) values.

Since POC primarily reflects neuromuscular coordination and symmetry rather than absolute force generation [27,28], these findings may indicate a more rapid re-establishment of balanced muscle recruitment patterns. In contrast, bimaxillary patients demonstrated greater variability and a slower progression toward symmetry, in line with previous studies reporting that neuromuscular recovery after orthognathic surgery is progressive, individualized, and influenced by the extent of skeletal repositioning [29,31,36].

Impact values, which represent the expression of muscular force over time, supported this trend. Although Impact values decreased in all patients during the orthodontic and immediate postsurgical phases, monomaxillary patients—particularly those treated with maxillary surgery alone—showed a faster increase toward reference ranges during follow-up [24,25]. At the end of treatment, Impact values in the monomaxillary group were closer to commonly accepted physiological reference values (approximately 100 ± 15%·s) [27], whereas bimaxillary patients tended to remain slightly below this range.

In the context of orthognathic and orthodontic treatment, the IMPACT index is considered a global indicator of masticatory muscle performance, as it summarizes the functional contribution of the elevator muscles rather than isolated peak activity. Changes in IMPACT values may therefore reflect functional adaptation, neuromuscular reorganization, or recovery of muscular efficiency following treatment.

Similar findings have been reported in studies indicating that functional recovery following more extensive surgical repositioning may require longer adaptation periods and may not always reach preoperative or normative levels [22,23,24,25,29].

Electromyographic activity expressed in microvolts followed a comparable pattern. All patients showed signs of neuromuscular decompensation after the initiation of orthodontic treatment, without significant differences between surgical approaches. During the subsequent phases, monomaxillary patients demonstrated a more homogeneous recovery, whereas bimaxillary patients exhibited wider inter-individual variability. This variability has been extensively documented in the literature and reinforces the concept that neuromuscular adaptation after orthognathic surgery is highly patient-specific and modulated by multiple factors, including muscle morphology, skeletal pattern, and adaptive capacity [5,28,29].

Further distinctions emerged from subgroup analysis within the monomaxillary cohort. Patients treated with maxillary surgery alone showed higher POC and Impact values and a more rapid normalization of muscle activity compared with those treated with mandibular surgery alone. This observation may be related to the greater biomechanical and neuromuscular demands associated with mandibular repositioning, which directly alters muscle length–tension relationships and mandibular biomechanics [22,23,24,25]. Similar findings have been reported in studies evaluating mandibular advancement or setback procedures, where functional improvement was often delayed and heterogeneous across patients [4,5,16,28]. Nevertheless, these subgroup findings should be interpreted with caution, as the study was not specifically powered to detect differences within monomaxillary subgroups.

Electrokinesiographic data were consistent with the electromyographic findings. Maximum mandibular opening and protrusive movements improved after surgery in all groups; however, monomaxillary patients demonstrated a more stable recovery, approaching physiological values by the end of treatment. In agreement with previous investigations, some kinesiographic parameters appeared to require longer periods to normalize compared with electromyographic indices, confirming that different functional components of the stomatognathic system recover at different rates [29,36]. In line with the Results, differences in protrusive movements tended to decrease over time and were no longer statistically significant at final follow-up, while lateral movements did not differ between groups at any time point [38,39,40,41].

The lack of significant intergroup differences in lateral and protrusive mandibular movements indicates that both monomaxillary and bimaxillary surgical approaches allow comparable recovery of mandibular kinematics, supporting their clinical equivalence in restoring functional jaw mobility.

Overall, the present findings support the notion that neuromuscular adaptation after orthodontic–surgical treatment is not solely dependent on the correction of skeletal morphology, but is also influenced by the extent of surgical intervention and the complexity of occlusal reorganization. As previously suggested, severe morphological discrepancies are not necessarily associated with proportional functional impairment, and functional recovery does not occur uniformly across all neuromuscular parameters [4,26,29,36], underscoring the need for a multidimensional functional assessment. The interpretation of the findings was primarily based on clinically meaningful longitudinal patterns rather than on isolated statistically significant differences; consequently, several results should be regarded as trends of neuromuscular adaptation over time, acknowledging that multiple comparisons across different treatment phases may increase the risk of type I error.

These adaptation patterns suggest that bimaxillary surgery does not impair long-term neuromuscular function, but rather induces a more prolonged adaptation phase compared with monomaxillary surgery. Clinically, this supports the use of bimaxillary procedures when indicated, with the expectation of comparable functional outcomes at follow-up, albeit with different recovery dynamics.

The extent of surgical intervention primarily influences the timing and complexity of neuromuscular adaptation rather than the final functional outcome, with bimaxillary surgery requiring a longer reorganization phase before achieving neuromuscular patterns comparable to monomaxillary surgery.

From a methodological perspective, the longitudinal interpretation of the present findings should be approached with caution. Although repeated measurements were collected within the same subjects, the statistical analysis was not based on repeated-measures or mixed-effects models. Consequently, intra-subject correlation and formal time × group interactions were not modeled, and the observed temporal patterns should be regarded as descriptive rather than inferential. The subgroup comparison between maxillary-only and mandibular-only procedures should be interpreted with caution. This analysis was exploratory and underpowered, and it was not included in the original sample size calculation. Therefore, the observed differences should be regarded as hypothesis-generating rather than as evidence of distinct mechanistic pathways.

These results should not be interpreted as an indication to prefer one surgical approach over another, as the choice between monomaxillary and bimaxillary surgery remains primarily driven by skeletal, occlusal, and aesthetic requirements. Rather, the findings highlight the relevance of functional assessment as a complementary tool in the orthodontic–surgical workflow. The inclusion of a control group with physiological occlusion represents a strength of the present study, as it allowed interpretation of the postoperative neuromuscular recovery not only in relative terms over time, but also in relation to reference functional patterns observed in healthy subjects.

It is important to distinguish statistical significance from clinical relevance. Several statistically significant differences observed in the present study were numerically small and may fall within the range of normal physiological variability of electromyographic and kinesiographic parameters. As such, these differences should be interpreted cautiously, as they may represent statistically detectable but clinically modest effects rather than functionally meaningful changes.

For this reason, greater interpretative weight was given to consistent longitudinal trends across multiple parameters and treatment phases rather than to isolated statistically significant findings with limited numerical magnitude.

### Limitations

Several Limitations must be acknowledged.

First, patients were grouped according to the surgical approach rather than skeletal classification or severity of deformity. This reflects routine clinical decision-making but limits the ability to isolate the independent effect of surgical extent on neuromuscular outcomes.

Second, potential confounding factors such as magnitude of skeletal displacement, fixation technique, and duration of postsurgical orthodontic treatment were not included in the statistical analysis. Larger samples would allow multivariate modeling to better isolate the independent contribution of each variable. Additional limitations include the lack of stratification based on the magnitude of skeletal displacement, the possible influence of different surgical fixation techniques on neuromuscular recovery, and the absence of patient-reported outcomes assessing subjective masticatory function.

An additional limitation relates to multiple testing. Another limitation concerns the statistical approach. The use of independent-sample comparisons at each time point does not account for intra-subject correlation inherent to repeated measurements and limits the formal assessment of temporal interactions. Future studies with larger and more homogeneous samples should employ repeated-measures ANOVA or linear mixed-effects modeling to more robustly investigate time-dependent neuromuscular adaptations.

Finally, although evaluations were performed after completion of treatment and after a 2y follow up, longer-term follow-up is necessary to determine whether the observed neuromuscular differences persist over time and whether they influence long-term stability or relapse.

## 5. Conclusions

Within the limitations of the present study, the neuromuscular response to orthodontic–surgical treatment appeared to be associated with the surgical approach adopted.

Monomaxillary surgery, particularly maxillary-only procedures, was associated with a trend toward faster and more stable neuromuscular recovery, whereas bimaxillary surgery showed a slower and more variable adaptive pattern. These findings should be interpreted as associative observations rather than causal effects and do not support a preference for one surgical approach over another.

These results describe longitudinal associative patterns of neuromuscular adaptation across treatment phases, rather than definitive time-dependent effects. The observed differences should be interpreted in light of their magnitude and clinical relevance, acknowledging that some effects may be modest despite statistical significance.

Further longitudinal studies are required to clarify the long-term clinical relevance of these neuromuscular differences and their potential role in treatment stability and relapse prevention.

## Figures and Tables

**Table 1:** Percent overlapping coefficient (POC, %).

Percent Overlapping Coefficient (POC, %)		
Phase	Monomaxillary	Bimaxillary
T0	80.2 ± 3.5	79.6 ± 3.8
T1	75.1 ± 4.0	74.3 ± 4.2
T2	**80.4 ± 3.6** *	78.1 ± 4.1
T3	84.5 ± 3.3	81.8 ± 4.0
T4	**86.0 ± 3.2** *	82.9 ± 3.9

* Statistically significant difference vs. bimaxillary group (*p* < 0.05).

**Table 2:** Impact (µV·s).

Impact (µV·s)		
Phase	Monomaxillary	Bimaxillary
T0	5000 ± 800	4950 ± 820
T1	4000 ± 700	3900 ± 750
T2	2500 ± 600	2000 ± 500
T3	5800 ± 900	5200 ± 850
T4	6200 ± 900 *	5600 ± 850

* Statistically significant difference vs. bimaxillary group (*p* < 0.05).

**Table 3:** Muscular activity (µV).

Muscolar Activity (µV)		
Phase	Monomaxillary	Bimaxillary
T0	5000 ± 750	4950 ± 780
T1	4000 ± 700	3900 ± 720
T2	2500 ± 600	2100 ± 550
T3	5800 ± 850	5300 ± 820
T4	6200 ± 850 *	5700 ± 800

* Statistically significant difference vs. bimaxillary group (*p* < 0.05).

## Data Availability

The data supporting the findings of this study are available from the corresponding author upon reasonable request.

## Abbreviations

The following abbreviations are used in this manuscript:

EKG	Electrokinesiography
EMG	Electromyography
IMPACT	Integrated Muscle Performance Activity over Time
MVC	Maximum Voluntary Clench
POC	Percent Overlapping Coefficient
sEMG	Surface Electromyography
TENS	Transcutaneous Electrical Nerve Stimulation
TMJ	Temporomandibular Joint
µV	Microvolts
µV·s	Microvolt-seconds
